# Deep Learning for Automatic Image Segmentation in Stomatology and Its Clinical Application

**DOI:** 10.3389/fmedt.2021.767836

**Published:** 2021-12-13

**Authors:** Dan Luo, Wei Zeng, Jinlong Chen, Wei Tang

**Affiliations:** The State Key Laboratory of Oral Diseases and National Clinical Research Center for Oral Diseases & Department of Oral and Maxillofacial Surgery, West China College of Stomatology, Sichuan University, Chengdu, China

**Keywords:** deep learning, convolutional neural networks, transformer, automatic segmentation, stomatological image

## Abstract

Deep learning has become an active research topic in the field of medical image analysis. In particular, for the automatic segmentation of stomatological images, great advances have been made in segmentation performance. In this paper, we systematically reviewed the recent literature on segmentation methods for stomatological images based on deep learning, and their clinical applications. We categorized them into different tasks and analyze their advantages and disadvantages. The main categories that we explored were the data sources, backbone network, and task formulation. We categorized data sources into panoramic radiography, dental X-rays, cone-beam computed tomography, multi-slice spiral computed tomography, and methods based on intraoral scan images. For the backbone network, we distinguished methods based on convolutional neural networks from those based on transformers. We divided task formulations into semantic segmentation tasks and instance segmentation tasks. Toward the end of the paper, we discussed the challenges and provide several directions for further research on the automatic segmentation of stomatological images.

## Introduction

Imaging examinations, intraoral scanning, and other technologies are often required to assist diagnosis and treatment of diseases because of the complex structure of the oral and maxillofacial region and various types of diseases. Imaging examinations use dental X-rays, panoramic radiography, cone-beam computed tomography (CBCT), and multi-slice spiral computed tomography (MSCT). These are widely used in stomatology and produce large amounts of medical image data. Efficient and accurate processing of medical images is essential for the development of stomatology. The key task is image segmentation, which can realize the localization and the (qualitative and quantitative) analysis of lesions, help to design a treatment plan, and analyze the efficacy of the treatment. The traditional manual segmentation method is time-consuming and the segmentation effect depends on the experience of the doctor, leading to an unsatisfactory result. Therefore, the application of modern image segmentation technology to stomatology is very important.

Deep learning (DL) is a branch of machine learning and is a promising method of achieving artificial intelligence. Owing to the availability of large-scale annotated data and powerful computing resources, DL-based medical image segmentation algorithms have achieved excellent performance. These methods have successfully assisted the accurate diagnosis and minimally invasive treatment of brain tumors (1), retinal vessels (2), pulmonary nodules (3), cartilage, and bone (4). This paper reviews current DL-based medical image segmentation methods and their applications in stomatology. Existing automatic segmentation algorithms are classified according to the data source, the form of the automatic segmentation task, and the structure of the backbone network of the algorithm. The feasibility, accuracy, and application prospects of these algorithms are comprehensively analyzed, and their future research prospects are discussed.

## Stomatological Imaging Data Sources and Comparison

Common stomatological images can be categorized into five types: panoramic radiography, dental X-rays, CBCT, MSCT, and intraoral scanning (IOS). Each type is suitable for specific clinical applications, according to its unique imaging principles. Dental X-rays and panoramic radiography are mainly used for dental caries, alveolar bone resorption, and impacted teeth. CBCT is mainly used for the early diagnosis and comprehensive analysis of cracked teeth, diseases after root canal treatment, jaw lesions, and other diseases. CBCT can also assist the design of implant guides, orthodontic treatment, and maxillofacial disease treatment. MSCT is often conducted to assist the diagnosis, treatment, and postoperative efficacy analysis of soft and hard tissue lesions in the maxillofacial region. IOS is generally employed in chair-side digital restoration, digital orthodontics, and digital implant surgery. There is a structural overlap between dental X-rays and panoramic radiography because both produce 2D images. Without clinical experience in reading films, missed diagnoses and misdiagnoses may easily occur. Although CBCT and MSCT produce 3D images with clear layers, traditional empirical reading could also lead to missed diagnoses and misdiagnoses of early and minor lesions. Table 1 shows the imaging characteristics, advantages, and disadvantages of different types of data, together with the clinical application prospects of DL.

**Table 1:** The imaging characteristics, advantages, and disadvantages of different data types and the prospects for clinical application of deep learning.

**Data types**	**Imaging characteristics**	**Advantages**	**Disadvantages**	**Prospects for clinical application of deep learning**
Dental X-rays, panoramic radiography	2D	Easy to operate, low dose, fast imaging	Lack of 3D information	Assisting in diagnosing and screening diseases quickly and accurately. Reducing missed diagnosis and misdiagnosis.
CBCT	3D	High spatial resolution, short exposure time, low effective radiation dose and small metal artifacts	Low density resolution	1. Rapid and accurate segmentation of teeth or lesions can assist early diagnosis and reduce missed diagnosis and misdiagnosis. 2. Rapid and accurate dentition can meet the needs of implant guide plate design and orthodontic treatment design.
MSCT	3D	High density resolution	Low spatial resolution, long exposure time, high effective radiation dose and large metal artifacts	1. Reducing the missed diagnosis and misdiagnosis. 2. Automatically segmenting lesions or normal structures, can be used in intraoperative interaction, registration, and treatment design.
IOS	Surface 3D data	Obtaining the 3D data of tooth and soft and hard tissue surface in real time	Lack of internal data within soft and hard tissue	1. Pursing for segmenting tooth accurately. 2. Fast data-processing, obtaining results in a few seconds.

## Automatic Segmentation Algorithms for Medical Images

**Figure 1:**
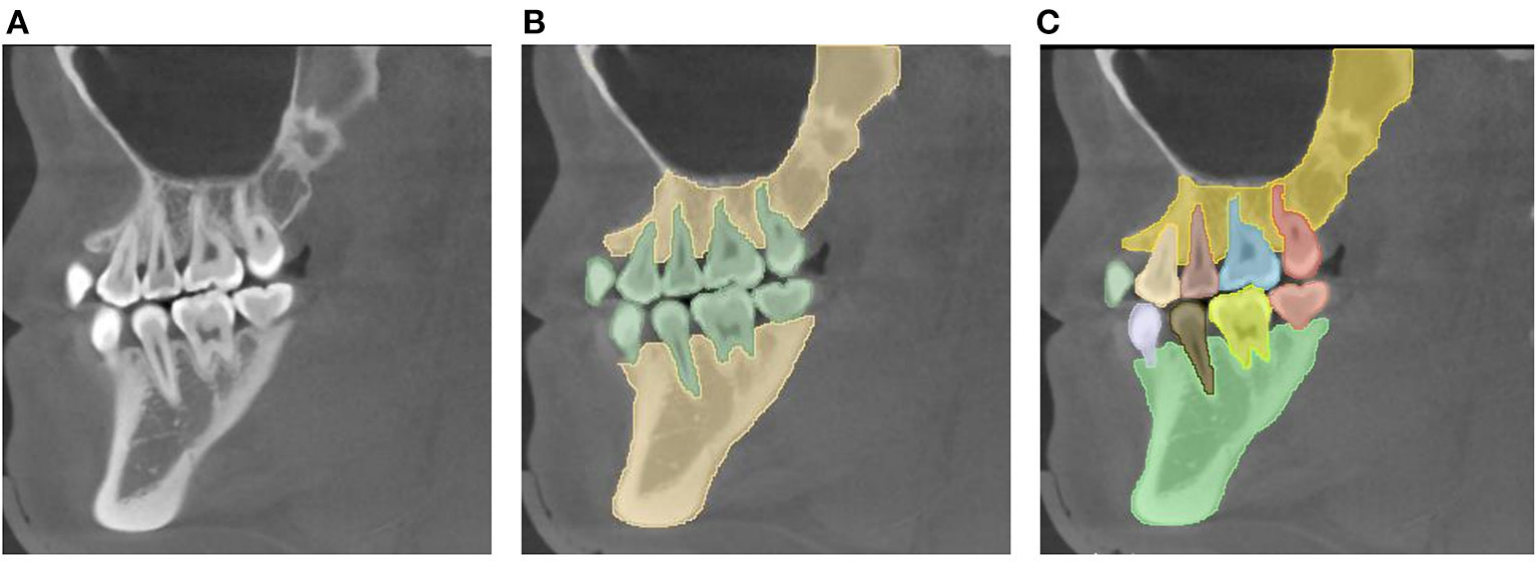
Task definitions for automatic image segmentation. **(A)** The original image. **(B)** Semantic segmentation: it is required to segment the teeth, jaws, and background, without the need to distinguish the individuals in the category “Tooth” or “Jaw.” **(C)** Instance segmentation: not only the category label is required, but also the instance label among the same class is needed, i.e., separating the individuals in the category “Tooth” or “Jaw”.

Image segmentation aims to simplify or change the representation of images, making them easier to understand and analyze. Image segmentation can be divided into the semantic segmentation task and the instance segmentation task. The semantic segmentation task focuses on differences between categories, whereas the instance segmentation task focuses on differences between individuals (Figure 1). In the semantic segmentation task, it is required to separate the teeth, jaws, and background, without distinguishing between individuals in each category (“Tooth” or “Jaw).” Conversely, in the instance segmentation task, both the category label and the instance label (within the class) are required; that is, the individuals in each category (“Tooth” or “Jaw)” must be distinguished.

Traditional image segmentation algorithms (5–7) cannot be directly applied to complex scenes because of the limitations of their manually designed features. The emergence of DL has made it possible to segment medical images efficiently and effectively. Segmentation algorithms based on convolutional neural networks (CNNs) have already become the *de facto* standard in image segmentation tasks. Their excellent segmentation ability has been demonstrated experimentally and theoretically and can be further applied to medical images. In addition to the popularity of CNNs, the transformer structure (8), originating from the field of natural language processing, has become an active research topic in computer vision because of its excellent long-term modeling ability. Therefore, according to these different types of backbone networks, we divide automatic image segmentation methods into CNN-based methods and transformer-based methods.

**Figure 2:**
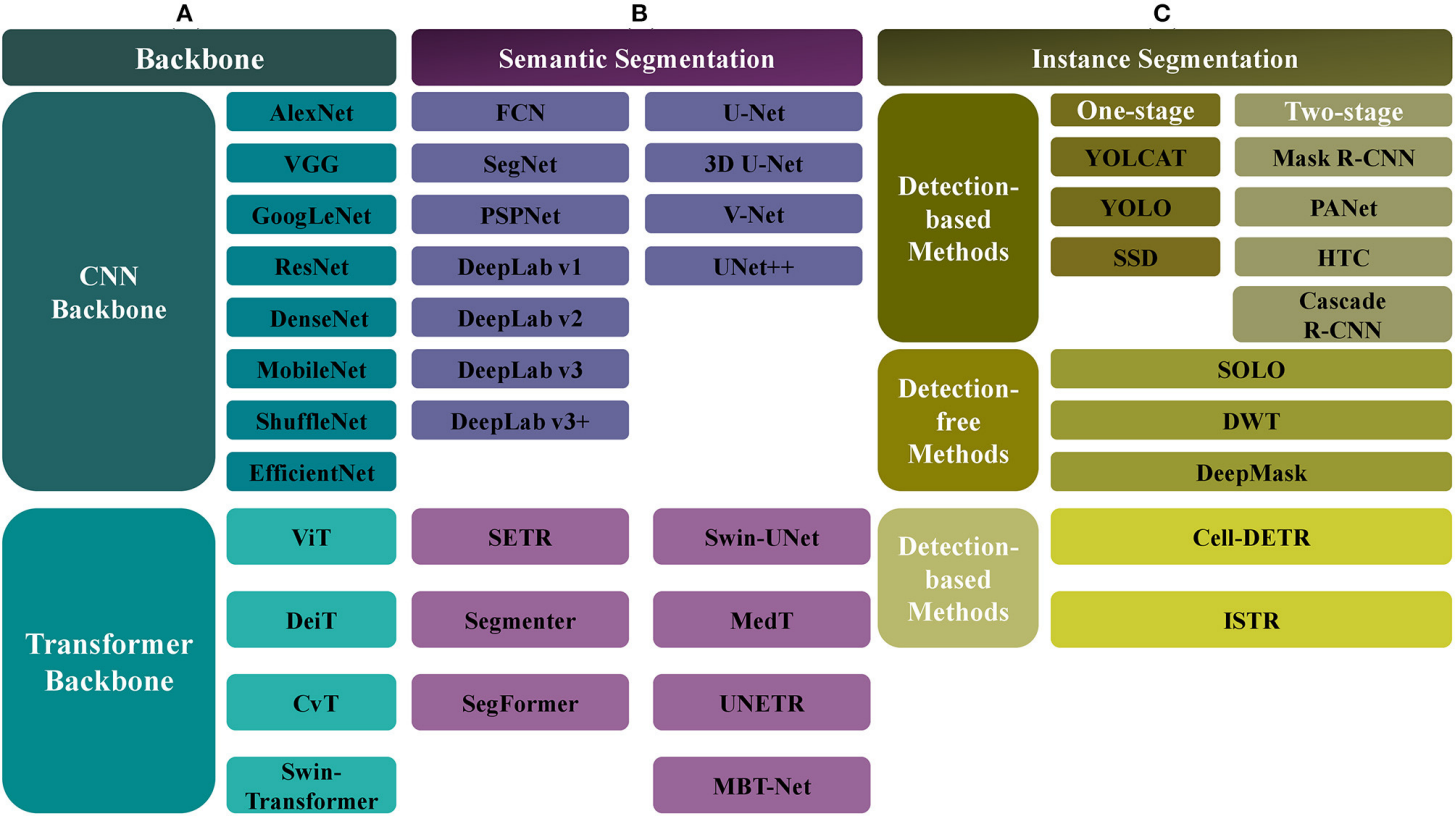
The overview of automatic segmentation algorithms. **(A)** For the backbone network, there are CNN-based and Transformer-based methods, the former includes AlexNet, VGG, GoogLeNet, ResNet, DenseNet, MobileNet, ShuffleNet, and EfficientNet, and the latter includes ViT, Data-efficient image Transformers (DeiT), Convolutional vision Transformer (CvT), and Swin-Transformer. **(B)** For the semantic segmentation, the CNN-based methods include FCN, SegNet, PSPNet, DeepLab (v1, v2, v3, v3+), UNet, VNet, and UNet++, and the Transformer-based methods include SETR, Segmenter, SegFormer, Swin-UNet, Medical Transformer (MedT), UNETR, MBT-Net, TransUNet, and TransFuse. **(C)** The instance segmentation task also can be categorized into CNN-based and Transformer-based methods. Meanwhile, it can be divided into the detection-based and the detection-free instance segmentation methods, the former is divided into the single-stage (YOLCAT, YOLO, and SSD) and two-stage methods (Mask R-CNN, PANet, Cascade R-CNN, and HTC), and the latter includes SOLO, DWT, and DeepMask. The Transformer-based methods, such as cell-DETR, ISTR, belong to detection-based methods.

**Figure 3:**
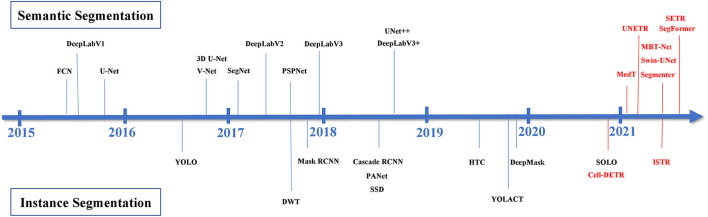
The development of automatic image segmentation. The black represents CNN-based methods and the red shows Transformer-based methods.

We have collected and summarized about 30 articles on image segmentation tasks. An overview of these methods is shown in Figure 2, and their evolution over time is depicted in Figure 3.

### Principles and Development of CNNs and Transformers

#### CNNs

The main ingredients of a modern CNN are the convolution layer, nonlinear activation layer, pooling layer, and fully connected layer, of which the convolution layer is the core component. The main principles of the convolution layer are its local receptive fields and weights-sharing strategy; the first refers to the limited range of data within a sliding window, and the second refers to the shared parameters of convolution kernels despite the sliding windows. The pooling layer can reduce the resolution of extracted features, to reduce the amount of calculation, and select the most robust features to prevent overfitting. The fully connected layer refers to the connection between all nodes in two adjacent layers; such a layer can realize the integration and mapping of input features and is usually used to output classification results. The nonlinear activation layer provides nonlinearity to the neural network so that it can approximate all continuous functions.

AlexNet (9), an early CNN model, adopted the ReLU activation function to accelerate network convergence and the dropout technique to prevent overfitting. VGG (10) achieved better performance than AlexNet by replacing the large 5 × 5 convolution kernel with two continuous 3 × 3 convolution kernels and increasing the network depth. GoogLeNet (11) used the Inception module to increase the width of the network while reducing the number of parameters. Its subsequent version (12) improved performance by convolution decomposition, batch normalization, label smoothing, and other techniques. ResNet (13) solved the problem of network degradation by using skip connections and has been one of the most popular feature extractors in many vision tasks. DenseNet (14) made full use of extracted features by establishing dense connections between different layers. Moreover, lightweight models [e.g., MobileNet (15) and ShuffleNet (16)] and models designed by neural architecture search (NAS) [e.g., EfficientNet (17)] have already received widespread attention in the DL community.

#### Transformers

The transformer structure, which originated from natural language processing (18–20), has recently attracted substantial attention from the vision community. The transformer (18) proposed the multi-head self-attention module and a feedforward network to model long-term relationships within input sequences; it also has an enhanced ability for parallel computing. Witnessing the power of transformers in natural language processing, some pioneering studies (21–24) have successfully applied it to image classification, object detection, and segmentation tasks.

Vision Transformer (ViT) (21) split an image into patches and directly fed these patches into the standard transformer with positional embeddings, demonstrating that learning from large-scale data is better than inductive bias. Data-efficient image Transformers (DeiT) (22) achieved better performance by more careful training strategies and token-based extraction. Convolutional vision Transformer (CvT) (23) improved the performance and efficiency of ViT by introducing convolution into the ViT architecture. This was accomplished by two major modifications: the hierarchical transformer, containing convolutional token embedding, and a convolutional transformer block, using a convolutional projection. Swin-Transformer (24) is a hierarchical transformer whose features are calculated within a shifted window, providing higher efficiency by limiting the self-attention calculation to non-overlapping local windows and allowing cross-window connection; its computational complexity is linear with respect to image size. These features make Swin-Transformer compatible with a wide range of visual tasks, including image classification, object detection, and semantic segmentation.

### Common Algorithms for Semantic Segmentation

**Figure 4:**
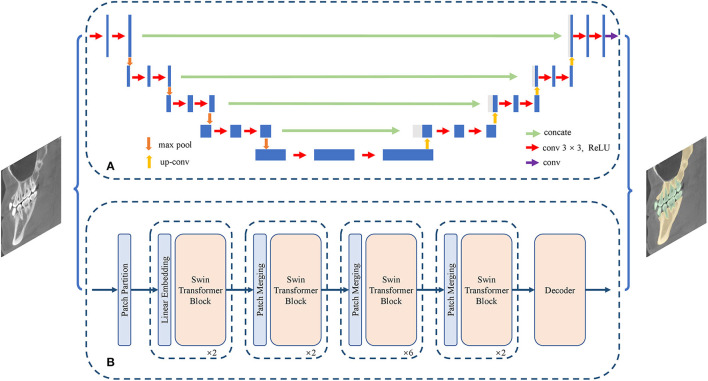
Structure of semantic segmentation network. **(A)** The CNN-based semantic segmentation approach, from UNet. **(B)** The Transformer-based semantic segmentation approach, from Swin-Transformer.

The aim of the semantic segmentation task is to assign a unique category label to each pixel or voxel in the image. Semantic segmentation can both identify and mark the boundaries of different categories, such as the boundaries of teeth and jaws. Depending on the backbone network used, CNN-based and transformer-based approaches have been developed (Figure 4). CNN-based semantic segmentation algorithms include FCN (25), SegNet (26), Pyramid Scene Parsing Network (PSPNet) (27), DeepLab (v1, v2, v3, v3+) (28–31), U-shaped Network (UNet) (32), 3D U-shaped Network (3D UNet) (33), V-shaped Network (VNet) (34), and UNet++ (35). Algorithms based on transformers include SEgmentation TRansformer (SETR) (36), Segmenter (37), SegFormer (38), Swin-UNet (39), Medical Transformer (MedT) (40), UNEt TRansformers (UNETR) (41), Multi-Branch Hybrid Transformer Network (MBT-Net) (42), TransUNet (43), and TransFuse (44).

#### CNN-Based Algorithms

As an iconic in image semantic segmentation, FCN (25) replaced all full connection layers with convolutional layers to predict the dense segmentation map. In contrast to FCN, SegNet (26) performed nonlinear upsampling according to the index of the max-pooling of the corresponding encoder, where the spatial information of the encoding stage was maintained. PSPNet (27) obtained the global context information by aggregating the context information, to improve the parsing performance for complex scenes.

The DeepLab series focused on enlarging the receptive field and integrating multi-scale feature information. DeepLabV1 (28) used dilated convolution and conditional random fields to obtain more informative feature maps. DeepLabV2 (29) featured the atrous spatial pyramid pooling module (ASPP), which performed the atrous convolution of different sampling rates to obtain multi-scale feature representation. DeepLabV3 (30) achieved the effect of atrous convolutions, multi-grid, and ASPP. DeepLabV3+ (31) used the encoder-decoder structure to perform segmentation tasks, and the depthwise separable convolution from Xception was introduced into the ASPP module.

UNet (32) was one of the most influential segmentation models dedicated to biomedical fields. Compared with FCN, its major contributions lay in its U-shaped symmetric network and an elastic deformation-based data augmentation strategy. The U-shaped network consisted of symmetric compression paths and expansion paths, and the elastic deformation effectively simulated the normal changes in cell morphology. 3D UNet (33) implemented a 3D image segmentation task by replacing the 2D convolution kernel in UNet (32) with a 3D convolution kernel. VNet (34) used a new loss function, termed Dice loss, to handle the limited number of annotated volumes available for training. UNet++ (35) introduced a built-in ensemble of UNets of varying depths and had redesigned skip connections to enhance performance for objects of varying sizes.

#### Transformer-Based Algorithms

SETR (36) used a pure transformer to encode an image to a sequence of patches, without the need for a convolution layer or resolution reduction and showed the power of the transformer structure for segmentation tasks. In Segmenter (37), the global context relationship was established from the first layer and a pointwise linear decoder was employed to obtain the semantic labels. SegFormer (38) combined the hierarchical transformer structure with a lightweight multi-layer perceptron decoder, without the need for positional encoding or a complex decoder.

Swin-UNet (39) unified UNet with a pure transformer structure for medical image segmentation tasks, by feeding tokenized image blocks into the symmetric transformer-based U-shaped encoder-decoder architecture with skip connections, and local and global cues were fully exploited. The successful application of Swin-UNet to multi-organ and cardiac segmentation tasks demonstrated the potential benefits of the transformer structure to medical image segmentation. MedT (40) featured the gated axial-attention model, in which an additional control mechanism was introduced into the self-attention module. In addition, a local-global training strategy (LoGo) was proposed to further improve performance. UNETR (41) employed pure transformers as an encoder to capture global multi-scale information effectively. The effectiveness of UNETR in 3D brain tumors and spleen tasks (CT and MRI modalities) was validated by experiments on the MSD dataset. MBT-Net (42) applied a multi-branch hybrid transformer network, which was composed of a body branch and an edge branch, to the corneal endothelial cell segmentation task. Other transformer-based methods for medical image segmentation include TransUNet (43) and TransFuse (44).

### Common Algorithms for Instance Segmentation

Depending on the backbone network used, instance segmentation methods can also be categorized into CNN-based and transformer-based methods. In addition, from the perspective of algorithms, instance segmentation methods can be divided into detection-based and detection-free methods. Detection-based methods can be regarded as extensions of object detection: they obtain bounding boxes by object detection methods and then perform segmentation within the bounding boxes. Moreover, the detection methods can be divided into single-stage and two-stage methods. Single-stage methods include You Only Look At Coefficient Ts (YOLCAT) (45), You Only Look Once (YOLO) (46), and Single Shot MultiBox Detector (SSD) (47). Two-stage methods include Mask R-CNN (48), PANet (49), Cascade R-CNN (50), and hybrid task cascade (HTC) (51). Detection-free methods first predict the embedding vector and then group the corresponding pixel points into a single instance by clustering; examples of such methods include Segmenting Objects by Locations (SOLO) (52), Deep Watershed Tranform (DWT) (53), and DeepMask (54).

**Figure 5:**
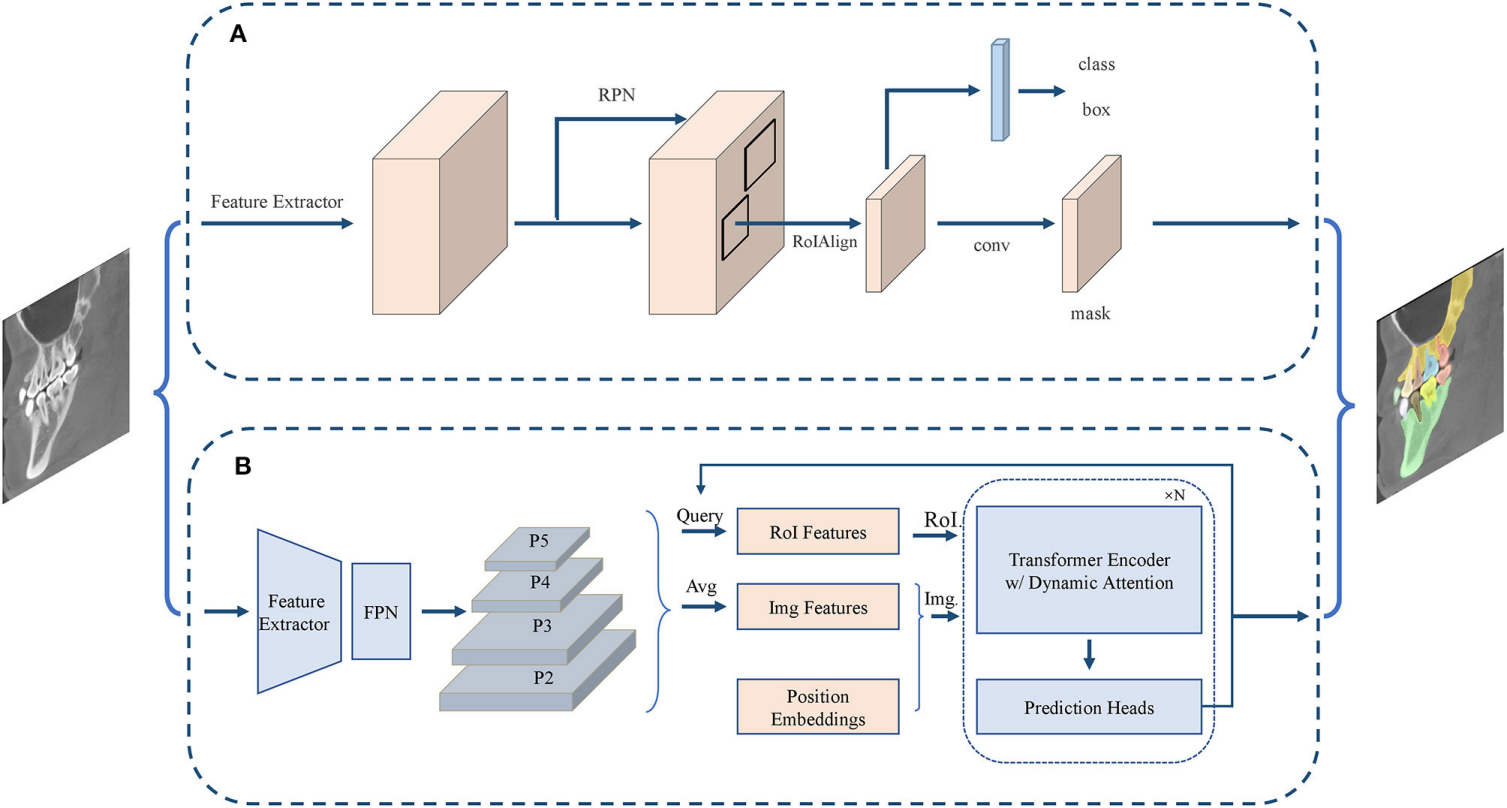
Structure of instance segmentation network. **(A)** The CNN-based instance segmentation approach, from Mask R-CNN. **(B)** The Transformer-based instance segmentation approach, from ISTR.

Most existing transformer-based instance segmentation algorithms are built on a detection method, DETR (55), so they belong to the class of detection-based methods; such methods include Cell-DETR (56) and ISTR (57). We have collected 12 articles on instance segmentation tasks; the overall development is shown in Figure 5.

#### CNN-Based Algorithms

Detection-based instance segmentation methods follow the principle of detecting first and then segmenting. The performance of such methods is heavily dependent on the performance of the object detector, and so a better detector would improve the quality of instance segmentation. As discussed above, detection methods can be divided into single-stage and two-stage methods. A typical example of a single-stage method is YOLCAT (45), which first generated multiple prototype masks, and then used the generated coefficient to combine prototype masks, to formulate the object detection and segmentation results. In addition, a popular single-stage object detector [e.g., YOLO (46)] could accomplish the instance segmentation task by adding the same mask branch. A typical example of a two-stage method is Mask R-CNN (48), which used RoIAlign for feature alignment and added an object mask prediction branch to Faster R-CNN (58). PANet (49) further aggregated the underlying and high-level features on the basis of Mask R-CNN and performed fusion operations by adaptive feature pooling for subsequent prediction. Cascade R-CNN (50) achieved the purpose of continuously optimizing the prediction results by cascading several detection networks with different IoU thresholds. HTC (51) had a multi-task and multi-stage hybrid cascade structure and incorporated a branch for semantic segmentation to enhance spatial context.

Detection-free instance segmentation methods learn the affinity relation by projecting each pixel onto embedding space, pushing pixels of different instances apart, and pulling pixels of the same instance closer in the embedding space; finally, a postprocessing step such as grouping can formulate the instance segmentation result. SOLO (52) was an end-to-end detection-free instance segmentation method, which could directly map the original input image to the required instance mask, eliminating the postprocessing requirements in detection. DWT (53) combined the traditional watershed transform segmentation algorithm with a CNN to perform instance segmentation. DeepMask (54) simultaneously generated a mask, indicating whether each pixel on a patch belongs to an object, and an objectiveness score, indicating the confidence of an object located at the center of the patch. Compared with detection-based instance segmentation methods, the performance of these detection-free methods is limited, and there is scope for improvement.

#### Transformer-Based Algorithms

Applying the transformer structure to instance segmentation tasks is a relatively new research area. Cell-DETR (56) was one of the first methods to apply the transformer structure to instance segmentation tasks on biomedical data, achieving performance comparable with that of the latest CNN-based instance segmentation methods, but having a smaller number of parameters and a simpler structure. ISTR (57), an end-to-end instance segmentation framework, predicted low-dimensional mask embeddings and assigned them with ground-truth mask embeddings to compute the set loss, achieving a significant performance gain for instance segmentation tasks by conducting a recurrent refinement strategy.

### Characteristics of Automatic Segmentation Algorithms

The characteristics of semantic segmentation algorithms are shown in Table 2, and those of instance segmentation algorithms are shown in Table 3. The code and the data of works of literature are shown in Appendix.

**Table 2:** Features of semantic segmentation algorithms.

**Models**	**Features**
FCN (25)	The first full convolution network in semantic segmentation task. Ignoring the global context information and having a relatively high usage of GPU memory.
SegNet (26)	Improving the segmentation performance at boundary, reducing the number of model parameters and calculation cost
PSPNet (27)	Taking the global context information into consideration, improving the segmentation of small objects and co-occurrent categories.
DeepLab series (28–31)	V1: enlarging the receptive field by atrous convolution. V2: obtaining multi-scale feature by ASPP module. V3: exploring the effect of atrous convolutions, multi-grid, atrous spatial pyramid pooling, useful for small objects. V3+:utilizing the decoder module to refine the segmentation results especially along object boundaries, which is a faster and stronger encoder-decoder network.
UNet (32), 3D UNet (33)	It is extremely suitable for segmenting medical images and can train from small-scale dataset with dedicated data augmentation.
VNet (34)	It is a variant of UNet and suitable for 3D image analysis.
UNet++ (35)	An advanced UNet structure, improving the performance on objects of varying size by unifying a set of UNet with different depth.
SETR (36)	A novel and accurate Transformer-based model on semantic segmentation task, without the need for convolution layer and resolution reduction.
Segmenter (37)	Applying Transformer structure to obtain global context information and achieving SOTA performance on ADE20K dataset.
SegFormer (38)	Simplifying the design of Transformer-based model, a lightweight multilayer perceptron decoder is proposed to avoid the complex design of decoder, without the need for positional encoding.
Swin-UNet (39)	A combination of UNet and Swin-Transformer, which is carefully designed for medical image segmentation, achieving high performance with small number of parameters.
MedT (40)	A Transformer-based medical image segmentation network without pre-training.
UNETR (41)	Effectively capturing the global and multi-scale information and achieving high performance on 3D brain tumors and spleen tasks.
MBT-Net (42)	Fully exploiting the global and local context information by Transformer and CNN respectively and achieving high performance on segmenting corneal endothelial cells.
TransUNet (43)	It combines the advantages of UNet and Transformer structure to make a strong method on many medical applications including multi-organ segmentation and cardiac segmentation tasks.
TransFuse (44)	It combines Transformers and CNNs in a parallel style, capturing both global and local information respectively, obtaining better results on both 2D and 3D medical image sets including polyp, skin lesion, hip, and prostate segmentation.

**Table 3:** Features of instance segmentation algorithms.

**Models**	**Features**
YOLACT (45)	A real-time instance segmentation method, which achieves the mAP of 29.8% and reaches 33 fps on MSCOCO dataset.
YOLO (46)	It takes object detection tasks as a regression problem to spatially split bounding boxes and class probabilities, reaching very high speed on many tasks while having a comparable mAP.
SSD (47)	A fast object detection method that predicts bounding box location by regression and object class by classification, reaching faster speeds comparing to Faster-RCNN, without the need for bounding box proposal and pixel/feature resampling.
Mask RCNN (48)	Adding a mask branch to the detection Fast R-CNN, proposing RoIAlign for feature alignment.
PANet (49)	It proposes a new feature fusion strategy for multi-scale features and obtains the winner in the COCO 2017 Challenge Instance Segmentation task and the 2nd place in Object Detection task without large-batch training.
Cascade-RCNN (50)	Continuously optimizing the prediction results by cascading several detection networks with different IoU thresholds.
HTC (51)	Proposing a multi-task and multi-stage hybrid cascade structure and achieve high performance on many tasks.
SOLO (52)	An end-to-end detection-free instance segmentation method
DWT (53)	Combining the traditional watershed transform algorithm with the CNN model
DeepMask (54)	An earlier instance segmentation method, relatively low performance.
Cell-DETR (56)	The first Transformer-based instance segmentation method for biomedical data and SOTA performance.
ISTR (57)	It is the first end-to-end Transformer-based framework in instance segmentation task, predicting low-dimensional mask embeddings, and then matching with ground truth mask embeddings for loss computing.

## Clinical Application of Automatic Image Segmentation in Stomatology

The segmentation of teeth, jaws and their related diseases is usually considered as a preprocessing step to complete tooth matching (59–62), tooth numbering (63–65), automatic marking of important anatomical markers, in addition to the intelligent diagnosis, classification, and prediction of diseases. Traditional methods for stomatological image segmentation include region-based (66), threshold-based (67), clustering-based (68), edge tracking (69), and watershed (8) methods. With the development of DL, many DL-based methods for stomatological image segmentation have been developed; these mainly focus on teeth, jaws, and their related diseases.

### Application to Teeth and Related Diseases

Automatic segmentation of teeth in stomatological images can contribute to the location of supernumerary teeth and impacted teeth, as well as digital restoration, digital orthodontics, and digital implant surgery. Automatic segmentation of caries and other related lesions is helpful for the early diagnosis of caries, particularly those that are easily missed, such as hidden caries and adjacent caries. At present, the types of medical image data that are commonly used for the segmentation of teeth and related lesions include panoramic radiography, CBCT, dental X-rays, and IOS.

Semantic segmentation is the most common type used for the DL-based automatic segmentation of teeth and their related diseases. This paper reviews 11 articles on semantic segmentation (Table 4), finding that semantic segmentation can mark the boundary between teeth and jaws, but the boundary is unclean, particularly for a malposed tooth that overlaps adjacent teeth. However, instance segmentation can distinguish different teeth in six relevant articles (Table 5). Compared with semantic segmentation, instance segmentation is better for marking the boundary of each tooth, but there are still some problems, such as the loss of data detail and small sample size, which may affect the accuracy of segmentation.

**Table 4:** Semantic segmentation in teeth and related diseases.

**Study**	**Year**	**Algorithm**	**Image type**	**Images total**	**Outcome metrics**	**Performance**
Wirtz (70)	2018	UNet	Panoramic	24	Accuracy, Specificity, Precision, Recall, F1-Score, DSC	0.818, 0.799, 0.790, 0.827, 0.803, 0.744
Koch (71)	2019	UNet	Panoramic	1500	DSC	0.934
Sivagami (72)	2020	UNet	Panoramic	1171	Accuracy, Specificity, Precision, Recall, F1-Score, DSC	0.97, 0.95, 0.93, 0.94, 0.93, 0.94
Choi (73)	2016	FCN	dental X-ray	475	F1-score	0.74,
Cui (74)	2021	ToothPix	Panoramic	1500	IOU, Accuracy, Specificity, Precision, Recall, F1-score	0.9042, 0.9808, 0.9852, 0.9407, 0.9591, 0.9486
Zakirov (75)	2018	VNet	CBCT	517	IOU, Accuracy	0.963, 0.96
Chen (76)	2020	FCN+MWT	CBCT	25	DSC, Jaccard, RVD, ASSD	0.936, 0.881, 0.072, 0.363 mm
Lee (77)	2020	CNN	CBCT	102	DSC, Recall, Precision	Validation set: 0.938, 0.952, 0.924; Testing set: 0.918, 0.932, 0.904
Rao (78)	2020	UNet+DCRF	CBCT	110	VD, DSC, ASSD, MSSD	18.86 mm^3^, 0.9166, 0.25 mm, 1.18 mm
Ezhov (79)	2019	VNet	CBCT	935	IOU, ASD	0.94, 0.17 mm
Zanjani (80)	2019	PointCNN	IOS	120	IOU	0.94

**Table 5:** Instance segmentation in teeth and related diseases.

**Study**	**Year**	**Algorithm**	**Image type**	**Images total**	**Outcome metrics**	**Performance**
Jader (81)	2018	mask RCNN	Panoramic	193	Accuracy, Specificity, Precision, Recall, F1-score	0.98, 0.99, 0.94, 0.84, 0.88
Silva (65)	2020	Mask RCNN, HTC, ResNeSt, PANet (best)	Panoramic	1,500	Accuracy, specificity, precision, recall, F1-Score	PANet: 0.967, 0.987, 0.944, 0.891, 0.916
Gurses (82)	2020	Mask RCNN+ SURF	Panoramic	580	Jaccard, Precision, Recall, F1-score, Rank-1 accuracy	0.82, 0.93, 0.91, 0.95, 0.8039
Wu (83)	2020	GH + BADice-DenseASPP-UNet + LO	CBCT	20	DSC, ASD, FA, DA	0.962, 0.122, 0.991, 0.995
Cui (84)	2019	ToothNet	CBCT	20	DSC	0.9264
Zanjani (85)	2021	Mask-MCNet	IOS	164	mIOU, mAP, mAR	0.98, 0.98, 0.97

#### Semantic Segmentation in Teeth and Related Diseases

For 2D images, different models can be trained to segment different areas, such as all teeth (70–72) or adjacent caries (73), depending on the artificially defined foreground. Wirtz (70), Koch (71), and Sevagami (72) all used the UNet network for the automatic segmentation of teeth from panoramic radiography. Wirtz et al. (70) also used the method to segment teeth in complex cases such as tooth loss, defect, filling, and fixed bridge restoration, achieving a Dice similarity coefficient (DSC) of 0.744 on their dataset. Koch et al. (71) proved that UNet improves the segmentation performance by exploiting data symmetry, an ensemble of the network, test-time augmentation, and bootstrapping; they measured a DSC of 0.934 on the dataset created by Silva (86). Sevagami et al. (72) believed that UNet could work well without dense connections, residual connections, or the Inception module; the DSC on the dataset obtained from Ivisionlab (81) was 0.94. Cui et al. (74) used the generative adversarial network to exploit comprehensive semantic information for tooth segmentation, with an IoU of 0.9042 on the LNDb dental dataset. Choi et al. (73) first aligned the teeth horizontally, generated the probability map of dental caries in periapical images with FCN, then extracted the crowns, and finally refined the caries probability map, to achieve automatic detection and segmentation of adjacent dental caries. The F1-score was 0.74 on their own dataset. These applications all perform semantic segmentation of 2D images; the only differences are the artificial definition of the foreground and the choice of semantic segmentation model.

Most 3D images of teeth originate from CBCT data, and semantic segmentation of these 3D images requires a 3D semantic segmentation network, such as VNet (75), multi-task 3DFCN and marker-controlled Watershed transform (MWT) (76), modified UNet (77), and the symmetric fully convolutional residual network with DCRF (78). Ezhov et al. (79) proposed a coarse-fine network structure to refine the volumetric segmentation of teeth, with an IoU of 0.94. The segmentation results can be used for applications such as tooth volume prediction (75), panoramic reconstruction (75), digital orthodontic simulation (76), and dental implant design (77).

The gingival tissue cannot be shown on the panoramic radiography or CBCT image, but it is very important for clarifying the relationship between the tooth and gingiva for digital restoration, implant, and orthodontics. For this reason, another imaging method has emerged in stomatology, namely, IOS, which can obtain real-time 3D data (which are point cloud data) of teeth and soft tissues. Zanjani et al. (80) proposed an end-to-end learning framework for semantic segmentation of individual teeth and gingivae from IOS data. This method was based on PointCNN; it used a non-uniform resampling mechanism and a compatible loss weighting to improve performance; it achieved an IoU of 0.94 on the own dataset of the authors.

The performance of the above methods is shown in Table 4.

#### Instance Segmentation in Teeth and Related Diseases

Instance segmentation can mark both the boundaries between different categories in the image, such as the boundaries between teeth and jaws and the boundaries of different individuals in the same category, such as the boundaries between different teeth.

Tooth instance segmentation from panoramic radiography is a common task in dentistry. Using the Mask R-CNN algorithm, Jader et al. (81) performed instance segmentation of teeth from panoramic images, using the transfer learning strategy to solve the problem of insufficient annotated data, and proposing a data augmentation method by separating the teeth; they achieved accurate segmentation, with an F1-score of 0.88 on the dataset (86). Silva et al. (65) analyzed the segmentation, detection, and tooth number performance of four end-to-end deep neural network frameworks, namely Mask R-CNN, PANet, HTC, and ResNeSt, on a challenging panoramic radiography dataset. Of these algorithms, PANet achieved the best segmentation performance, with an F1-score of 0.916 on the UFBA-UESC Dental Images Deep dataset. Gurses et al. (82) proposed a method for human identification from panoramic dental images using Mask R-CNN and SURF; they used two datasets [DS1: part of the dataset from (81), DS2: their own dataset], achieving an F1-score of 0.95.

The tooth structure from 3D data is clearer, which is an important clinical advantage in instance segmentation of teeth. Wu et al. (83) used a two-stage deep neural network, which included a global stage (heatmap regression UNet) to guide the localization of tooth ROIs together with a local stage (ROI-based DenseASPP-UNet) for fine segmentation and classification, to perform tooth instance segmentation from CBCT; they achieved a DSC of 0.962 on their own dataset. Cui et al. (84) proposed a two-stage automatic instance segmentation method (ToothNet), based on a deep CNN for CBCT images, which obtained a good result, with a DSC of 0.9264 on their own dataset. It exploited a novel learned edge map, similarity matrix, and the spatial relations between different teeth.

Tooth instance segmentation with IOS is also an important research direction. Zanjani et al. (85) proposed a model named Mask-MCNet, for instance segmentation of teeth from IOS data. This model positioned each tooth by predicting its 3D bounding box, and simultaneously segmented points belonging to the individual tooth without using voxelization or subsampling techniques. The model could also preserve the fine detail in the data, enabling the highly accurate segmentation required in clinical practice, and obtaining results in just a few seconds of processing time. On their own dataset, the mIOU achieved was 0.98.

The performance of the above methods is shown in Table 5.

### Application in Jaws and Related Diseases

There are many types of jaw diseases; moreover, the number of benign and malignant samples is unbalanced, which may easily cause missed diagnoses and misdiagnoses. Minimally invasive and precise treatment of these diseases generally requires precise location of lesions through preoperative planning and accurate intraoperative image guidance. Patients with craniomaxillofacial malformations require intelligent 3D symmetry analysis. The analysis of postoperative efficacy requires both subjective evaluations by doctors and patients, in addition to the quantitative and objective evaluation of the progression and outcome of lesions. Precise segmentation of the jaw or related diseases is important for clinical diagnosis and treatment. In the past, manual segmentation of the jaw and its lesions was time-consuming and laborious. Since the development of DL, researchers have used DL methods to learn the features of the jaw and its lesions, realizing automatic segmentation. The main sources of medical image data used for automatic segmentation are panoramic radiography, CBCT, and MSCT.

Six relevant articles showed that current DL-based automatic segmentation methods for jaws and related diseases mainly focus on semantic segmentation (Table 6). There are two factors that affect the segmentation performance: (1) The space between the mandibular condyle and the temporal articular surface is very small and contains an articular disc, which often affects the accuracy of mandibular segmentation. (2) The segmentation accuracy of jaw or teeth in the occlusion and non-occlusion scenarios can be different because of the influence of the contact between upper and lower teeth.

**Table 6:** Semantic segmentation in the jaw.

**Study**	**Year**	**Algorithm**	**Image type**	**Images total**	**Outcome metrics**	**Performance**
Kong (87)	2020	UNet	Panoramic	2602	Accuracy, Jaccard, HD, PPS, Para(M)	0.9928, 0.9829, 8.32, 41.0, 0.92
Li (88)	2020	Deetal-Perio (based-Mask RCNN)	Panoramic	470	mAP, DSC (all), DSC (single), F1-score, Accuracy	Suzhou dataset: 0.826, 0.868, 0.778, 0.878, 0.884;Zhongshan dataset: 0.841, 0.852, 0.748, 0.454, 0.817
Egger (89)	2018	FCN-32s, FCN-16s, FCN-8s (best)	MSCT	20	DSC	FCN-8s: 0.9203
Zhang (90)	2018	UNet	CBCT, MSCT	CBCT(77), MSCT(30)	DSC, SEN, PPV	Midface: 0.9319, 0.9282, 0.9361, Mandible: 0.9327, 0.9363, 0.9293
Torosdagli (91)	2019	Tiramisu (based on UNet and DenseNET)	CBCT	50	DSC	0.9382
Lian (92)	2020	DTNet	CBCT, MSCT	CBCT(77), MSCT(63)	DSC, SEN, PPV	0.9395, 0.9424, 0.9368

For panoramic radiography, Kong et al. (87) adopted the UNet structure for rapid and accurate segmentation of the maxillofacial region, with an accuracy of 0.9928 on their own dataset. Li et al. (88) proposed the Deetal-Perio method to predict the severity of periodontitis from panoramic radiography. To calculate alveolar bone absorption, Deetal-Perio first segmented and indexed the individual tooth by using Mask R-CNN with a novel calibration method. It then segmented the contour of the alveolar bone and calculated a ratio of individual teeth, to represent the alveolar bone absorption. Finally, Deetal-Perio predicted the severity of periodontitis according to the ratios of all the teeth. They adopted two datasets, namely the Suzhou dataset and Zhongshan dataset, with DSCs of 0.868 and 0.852, respectively. Egger et al. (89) automatically segmented the mandible by using FCN and carefully evaluated the mandible segmentation algorithm. Ten CT datasets were included and three network architectures, namely, FCN-32s, FCN-16s, and FCN-8s, were used; FCN-8s achieved the best performance, with a DSC of 0.9203. Zhang et al. (90) introduced a context-FCN for joint craniomaxillofacial bone segmentation and landmark digitization; the DSCs of midface and mandible on their own dataset were 0.9319 and 0.9327, respectively. Torosdagli et al. (91) presented a new segmentation network for the mandible, based on a unified algorithm combining UNet and DenseNET. Compared with the most advanced mandible segmentation methods, this method achieved better performance on craniofacial abnormalities and disease states; the DSC was 0.9382 on their own CBCT dataset and 0.9386 on the MICCAI Head and Neck Challenge 2015 dataset. Lian et al. (92) introduced an efficient end-to-end deep network, the multi-task dynamic transformer network (DTNet), to perform concurrent mandible segmentation and large-scale landmark localization in one pass, for large-volume CBCT images. The network contributed to the quantitative analysis of craniomaxillofacial deformities. The DSC achieved was 0.9395 on the own dataset of the author.

The performance of the above methods is shown in Table 6.

## Trends and Future Work

### Integration and Improvement of Data Quality

As a consequence of the need to protect patient privacy and the right to be informed, there are not always enough cases to establish a large-scale dataset dedicated to the segmentation of stomatological images. Moreover, the collected data are usually taken from diverse hospitals and machines, which further increases the difficulty of formulating universal benchmarks. Therefore, methods to effectively integrate, store, and safely share these data are of vital importance and are urgently required. The establishment of a shared dento-maxillofacial database can help to solve this problem, to some extent. Differences in the data acquisition settings and conditions (such as exposure time) used in each hospital lead to variation in the quality of image data (such as contrast and signal-to-noise ratio), which affects the accuracy and robustness of image segmentation. Further study should focus on standardizing and normalizing image data and improving data quality.

Currently, most data used for image segmentation in stomatology are produced by a single modality (single CBCT or MSCT). In the future, multi-modality data of the same case can be employed collaboratively, to fully exploit the correlative and complementary essence among these modalities; this may further boost the performance.

### Model Design in the Fully Supervised Case

The most common methods of image segmentation in stomatology are built on top of a CNN. However, the transformer structure has been gradually emerging in the field of computer vision because of its global modeling ability. It has been applied to mandible segmentation by researchers, outperforming the current CNN-based model. Transformer-based methods have the potential to obtain satisfactory results in medical image segmentation in the future. In addition, how to reduce the number of model parameters while ensuring accuracy is an active research topic, which is particularly important for the deployment of the medical image segmentation model and the promotion of related technology in clinical settings.

### Model Design for Insufficient Data Annotation

The existing DL-based algorithm relies heavily on large-scale data to learn discriminative features, but the process of labeling stomatological data is time-consuming and labor-intensive; therefore, how to learn effectively from an insufficient and imperfect dataset is an active research topic. There are several ways to solve such problems. First, to reduce the burden of the time-consuming and labor-intensive annotation process, for complete annotation data, weakly supervised and semi-supervised methods can be adopted. Second, to handle the existence of noise in manual labeling, algorithms that learn from noisy labels can be employed. Third, to solve the problem that existing methods cannot be generalized to new categories, techniques such as transfer learning, domain adaptation, and few-shot learning can be considered. In addition, unsupervised learning and self-supervised learning could also be used to explore the structural properties inside the dental image itself, providing a better prior for downstream tasks.

### Interpretability of Deep Learning

Although existing DL methods have shown excellent performance in stomatology, they have not been widely promoted because of the limitations of DL. In addition to the high computing cost and the need for large-scale datasets, the “black box” characteristic of DL methods is the main factor that hinders their application. To gain the trust of doctors, regulators, and patients, a medical diagnostic system must be transparent, interpretable, and explicable. Ideally, it should explain the complete logic of how a decision is made. Therefore, research on interpretability is most urgently needed for the application of DL to clinical diagnosis and treatment.

### Clinical Application in Stomatology

First, from the perspective of what to segment, current studies focus mainly on teeth and teeth-related diseases, whereas little attention is paid to jaw and jaw-related diseases, particularly for soft tissue and related diseases. However, studies on the latter aspects have more clinical significance, so more studies of this type are required. Second, the accuracy of image segmentation is the key to whether the DL methods can be applied clinically. Therefore, more studies are needed to enhance the accuracy and precision of image segmentation, to promote its use in the clinic. Finally, the first step of digital surgical technologies, such as guide templates, surgical navigation, and augmented reality technology, is to segment important structures or lesions, for which the traditional manual segmentation method is currently mainly used. In the future, DL-based automatic segmentation methods could be integrated with these technologies, to assist clinical practice more accurately and efficiently.

## Conclusion

This paper comprehensively reviews automatic segmentation algorithms based on DL and introduces their clinical applications. The review shows that DL has great potential to segment stomatological images accurately, and this can further promote the transformation of clinical practice from experientialism to digitization, precision, and individuation. In the future, more research is needed to further improve the accuracy of automatic image segmentation and realize intelligence.

## Author Contributions

DL, WZ, JC, and WT contributed to the conception and design of the study. DL wrote the first draft of the manuscript. All authors contributed to manuscript revision, read, and approved the submitted version.

## Funding

This study was supported by Sichuan Province Regional Innovation Cooperation Project (2020YFQ0012).

## Conflict of Interest

The authors declare that the research was conducted in the absence of any commercial or financial relationships that could be construed as a potential conflict of interest.

## Publisher's Note

All claims expressed in this article are solely those of the authors and do not necessarily represent those of their affiliated organizations, or those of the publisher, the editors and the reviewers. Any product that may be evaluated in this article, or claim that may be made by its manufacturer, is not guaranteed or endorsed by the publisher.

## Appendix

**Table:** The code and the data of works of literature.

**Study**	**Algorithm**	**Code**	**Data**
Wirtz (70)	UNet	https://github.com/zhixuhao/unet	Their own dataset
Koch (71)	UNet	https://github.com/zhixuhao/unet	The dataset created by Gil Silva
Sivagami (72)	UNet	https://github.com/zhixuhao/unet	https://github.com/IvisionLab/deep-dental-image
Choi (73)	FCN	https://github.com/shelhamer/fcn.berkeleyvision.org	Their own dataset
Cui (74)	ToothPix	Not available	lndb dental dataset: https://github.com/IvisionLab/dental-image
Zakirov (75)	VNet	https://github.com/mattmacy/vnet.pytorch	Their own dataset
Chen (76)	FCN+MWT	https://github.com/shelhamer/fcn.berkeleyvision.org	Their own dataset
Lee (77)	CNN	Not available	Their own dataset
Rao (78)	UNet+DCRF	https://github.com/zhixuhao/unet	Their own dataset
Ezhov (79)	VNet	https://github.com/mattmacy/vnet.pytorch	Their own dataset
Zanjani (80)	PointCNN	https://github.com/yangyanli/PointCNN	Their own dataset
Jader (81)	mask RCNN	https://github.com/matterport/Mask_RCNN	https://github.com/IvisionLab/deep-dental-image
Silva (65)	Mask RCNN, HTC, ResNeSt, PANet (best)	https://github.com/openmmlab/mmdetection	https://github.com/IvisionLab/deep-dental-image
Gurses (82)	Mask RCNN+ SURF	https://github.com/openmmlab/mmdetection	DS1: https://github.com/IvisionLab/deep-dental-image, DS2: their own data set
Wu (83)	GH + BADice-DenseASPP-UNet + LO	Not available	Their own dataset
Cui (84)	ToothNet	Not available	Their own dataset
Zanjani (85)	Mask-MCNet	Not available	Their own dataset
Kong (87)	UNet	https://github.com/zhixuhao/unet	Their own dataset
Li (88)	Deetal-Perio (based-Mask RCNN)	https://github.com/matterport/Mask_RCNN	Suzhou Dataset and Zhongshan Dataset
Egger (89)	FCN-32s, FCN-16s, FCN-8s (best)	https://github.com/shelhamer/fcn.berkeleyvision.org	Their own dataset
Zhang (90)	UNet	https://github.com/zhixuhao/unet	Their own dataset
Torosdagli (91)	Tiramisu (based on UNet and DenseNET)	https://github.com/zhixuhao/unet https://github.com/liuzhuang13/DenseNet	https://www.aicrowd.com/challenges/miccai-2021-hecktor
Lian (92)	DTNet	Not available	Their own dataset

## References

[1] Karayegen G Aksahin MF. Brain tumor prediction on MR images with semantic segmentation by using deep learning network and 3D imaging of tumor region. Biomed Signal Proces and Control. (2021) 66:102458. 10.1016/j.bspc.2021.102458

[2] Atli I Gedik O S. Sine-Net: a fully convolutional deep learning architecture for retinal blood vessel segmentation. Eng Sci Technol an Int J. (2021) 24:271–83. 10.1016/j.jestch.2020.07.008

[3] Messay T Hardie RC Tuinstra TR. Segmentation of pulmonary nodules in computed tomography using a regression neural network approach and its application to the lung image database consortium and image database resource initiative dataset. Med Image Anal. (2015) 22:48–62. 10.1016/j.media.2015.02.002 25791434

[4] Ambellan F Tack A Ehlke M Zachow S. Automated segmentation of knee bone and cartilage combining statistical shape knowledge and convolutional neural networks: data from the osteoarthritis initiative. Med Image Anal. (2019) 52:109–18. 10.1016/j.media.2018.11.009 30529224

[5] Hojjatoleslami SA Kruggel F. Segmentation of large brain lesions. IEEE Trans on Med Imaging. (2001) 20:666–9. 10.1109/42.932750 11465472

[6] Alsmadi MK. A hybrid Fuzzy C-Means and neutrosophic for jaw lesions segmentation. Ain Shams Eng J. (2018) 9:697–706. 10.1016/j.asej.2016.03.016

[7] Li H Sun G Sun H Liu W. Watershed algorithm based on morphology for dental X-ray images segmentation[C]//2012 IEEE 11th international conference on signal processing. IEEE. (2012) 2:877–80. 10.1109/ICoSP.2012.649172027295638

[8] Devlin J Chang MW Lee K Toutanova K. Bert: Pre-Training of Deep Bidirectional Transformers For Language Understanding. arXiv [Preprint]. arXiv:1810.04805 (2018).

[9] Krizhevsky A Sutskever I Hinton GE. Imagenet classification with deep convolutional neural networks. Adv Neural Inf Process Syst. (2012) 25:1097–105. 10.1145/3065386

[10] Simonyan K Zisserman A. Very Deep Convolutional Networks For Large-Scale Image Recognition. San Diego, CA: ICLR 2015 (2014). arXiv [Preprint]. arXiv:1409.1556 (2014).

[11] Szegedy C Liu W Jia Y Sermanet P Reed S Anguelov D. Going deeper with convolutions. In: Proceedings of the IEEE conference on computer vision and pattern recognition. Boston, MA, USA (2015).

[12] Szegedy C Vanhoucke V Ioffe S Shlens J Wojna Z. Rethinking the inception architecture for computer vision. In: Proceedings of the IEEE conference on computer vision and pattern recognition. Las Vegas, NV, USA (2016).

[13] He K Zhang X Ren S Sun J. Deep residual learning for image recognition. In: Proceedings of the IEEE conference on computer vision and pattern recognition. Las Vegas, NV, USA (2016).

[14] Huang G Liu Z Van Der Maaten L Weinberger K Q. Densely connected convolutional networks. In: Proceedings of the IEEE conference on computer vision and pattern recognition. Honolulu, HI, USA (2017).

[15] Yu D Xu Q Guo H Zhao C Lin Y Li D. An efficient and lightweight convolutional neural network for remote sensing image scene classification. Sensors. (2020) 20:1999. 10.3390/s20071999 32252483 PMC7181261

[16] Zhang X Zhou X Lin M Sun J. Shufflenet: an extremely efficient convolutional neural network for mobile devices. In: Proceedings of the IEEE conference on computer vision and pattern recognition. Salt Lake City, UT, USA (2018).

[17] Tan M Le Q. Efficientnet: rethinking model scaling for convolutional neural networks. In: International Conference on Machine Learning. Long Beach, CA: PMLR (2019).

[18] Vaswani A Shazeer N Parmar N Uszkoreit J Jones L Gomez AN. Attention is all you need. In: Advances in neural information processing systems. Long Beach, CA: NIPS (2017).

[19] Dehghani M Gouws S Vinyals O Uszkoreit J Kaiser L. Universal Transformers. United States: ICLR 2018 (2018). arXiv [Preprint]. arXiv:1807.03819.

[20] Dai Z Yang Z Yang Y Carbonell J Le QV Salakhutdinov R. Transformer-xl:9 Attentive Language Models Beyond A Fixed-Length Context. Florence: Association for Computational Linguistics. arXiv [Preprint]. arXiv:1901.02860 (2019).

[21] Dosovitskiy A Beyer L Kolesnikov A Weissenborn D Zhai X Unterthiner T. An Image is Worth 16x16 Words: Transformers For Image Recognition At Scale. arXiv [Preprint]. arXiv:2010.11929 (2020).

[22] Touvron H Cord M Douze M Massa F Sablayrolles A Jégou H. Training data-efficient image transformers & distillation through attention. In: International Conference on Machine Learning. Long Beach, CA: PMLR (2021).

[23] Wu H Xiao B Codella N Liu M Dai X Yuan L Zhang L. CVT: Introducing Convolutions to Vision Transformers. arXiv [Preprint]. arXiv:2103.15808 (2021).

[24] Liu Z Lin Y Cao Y Hu H Wei Y Zhang Z. Swin Transformer: Hierarchical Vision Transformer Using Shifted Windows. arXiv [Preprint]. arXiv:2103.14030 (2021).

[25] Long J Shelhamer E Darrell T. Fully convolutional networks for semantic segmentation. In: Proceedings of the IEEE conference on computer vision and pattern recognition. Boston, MA, USA (2015). 10.1109/TPAMI.2016.2572683 27244717

[26] Badrinarayanan V Kendall A Cipolla R. Segnet: a deep convolutional encoder-decoder architecture for image segmentation. IEEE Trans Pattern Anal Mach Intell. (2017) 39:2481–95. 10.1109/TPAMI.2016.2644615 28060704

[27] Zhao H Shi J Qi X Wang X Jia J. Pyramid scene parsing network. In: Proceedings of the IEEE conference on computer vision and pattern recognition. Honolulu, HI: IEEE Computer Society (2017).

[28] Chen LC Papandreou G Kokkinos I Murphy K Yuille AL. Semantic Image Segmentation With Deep Convolutional Nets and Fully Connected Crfs. arXiv [Preprint]. arXiv:1412.7062 (2014). 28463186 10.1109/TPAMI.2017.2699184

[29] Chen LC Papandreou G Kokkinos I Murphy K Yuille AL. Deeplab: semantic image segmentation with deep convolutional nets, atrous convolution, and fully connected crfs. IEEE Trans Pattern Anal Mach Intell. (2017) 40:834–48. 10.1109/TPAMI.2017.2699184 28463186

[30] Chen LC Papandreou G Schroff F Adam H. Rethinking Atrous Convolution For Semantic Image Segmentation. arXiv [Preprint]. arXiv:1706.05587 (2017).

[31] Chen LC Zhu Y Papandreou G Schroff F Adam H. Encoder-decoder with atrous separable convolution for semantic image segmentation. In: Proceedings of the European conference on computer vision (ECCV). Munich: Springer (2018).

[32] Ronneberger O Fischer P Brox T. U-net: convolutional networks for biomedical image segmentation. In: International Conference on Medical image computing and computer-assisted intervention. Cham: Springer (2015).

[33] Çiçek Ö Abdulkadir A Lienkamp SS Brox T Ronneberger O. 3D U-Net: learning dense volumetric segmentation from sparse annotation. In: International conference on medical image computing and computer-assisted intervention. Cham: Springer (2016).

[34] Milletari F Navab N Ahmadi S A. V-net: fully convolutional neural networks for volumetric medical image segmentation. In: 2016 fourth international conference on 3D vision (3DV). Stanford, CA: IEEE (2016).

[35] Zhou Z Siddiquee MMR Tajbakhsh N Liang J. Unet++: a nested u-net architecture for medical image segmentation. In: Deep learning in medical image analysis and multimodal learning for clinical decision support. Springer, Cham (2018). 10.1007/978-3-030-00889-5_1 PMC7329239 32613207

[36] Zheng S Lu J Zhao H Zhu X Luo Z Wang Y. Rethinking semantic segmentation from a sequence-to-sequence perspective with transformers. In: Proceedings of the IEEE/CVF Conference on Computer Vision and Pattern Recognition. Salt Lake City, UT: IEEE (2021).

[37] Strudel R Garcia R Laptev I Schmid C. Segmenter: Transformer for Semantic Segmentation. arXiv [Preprint]. arXiv:2105.05633 (2021).

[38] Xie E Wang W Yu Z Anandkumar A Alvarez J M Luo P. SegFormer: Simple and Efficient Design For Semantic Segmentation With Transformers. arXiv [Preprint]. arXiv:2105.15203 (2021).

[39] Cao H Wang Y Chen J Jiang D Zhang X Tian Q. Swin-Unet: Unet-like Pure Transformer For Medical Image Segmentation. arXiv [Preprint]. arXiv:2105.05537 (2021).

[40] Valanarasu JMJ Oza P Hacihaliloglu I Patel VM. Medical Transformer: Gated Axial-Attention For Medical Image Segmentation. arXiv [Preprint]. arXiv:2102.10662 (2021).

[41] Hatamizadeh A Tang Y Nath V Yang D Myronenko A Landman B. Unetr: Transformers For 3d Medical Image Segmentation. arXiv [Preprint]. arXiv:2103.10504 (2021).

[42] Zhang Y Higashita R Fu H Xu Y Zhang Y Liu H. A Multi-Branch Hybrid Transformer Network for Corneal Endothelial Cell Segmentation. arXiv [Preprint]. arXiv:2106.07557 (2021).

[43] Chen J Lu Y Yu Q Luo X Adeli E Wang Y. Transunet: Transformers make strong encoders for medical image segmentation. arXiv [Preprint]. arXiv:2102.04306 (2021).

[44] Zhang Y Liu H Hu Q. Transfuse: Fusing Transformers and CNNs for Medical Image Segmentation. arXiv [Preprint]. arXiv:2102.08005 (2021).

[45] Bolya D Zhou C Xiao F Lee Y J. Yolact: real-time instance segmentation. In: Proceedings of the IEEE/CVF International Conference on Computer Vision. Seoul: IEEE (2019).

[46] Redmon J Divvala S Girshick R Farhadi A. You only look once: Unified, real-time object detection. In: Proceedings of the IEEE conference on computer vision and pattern recognition. Las Vegas, NV: IEEE Computer Society (2016).

[47] Liu W Anguelov D Erhan D Szegedy C Reed S Fu C Y Berg A C. SSD: single shot multibox detector. In: European conference on computer vision. Springer, Cham (2016).

[48] He K Gkioxari G Dollár P Girshick R. Mask R-CNN. In: Proceedings of the IEEE international conference on computer vision. Venice: IEEE Computer Society (2017).

[49] Liu S Qi L Qin H Shi J Jia J. Path aggregation network for instance segmentation. In: Proceedings of the IEEE conference on computer vision and pattern recognition. Salt Lake City, UT: Computer Vision Foundation / IEEE Computer Society (2018).

[50] Cai Z Vasconcelos N. Cascade R-CNN: delving into high quality object detection. In: Proceedings of the IEEE conference on computer vision and pattern recognition. Salt Lake City, UT: (2018). Computer Vision Foundation / IEEE Computer Society (2018). p. 6154–62.

[51] Chen K Pang J Wang J Xiong Y Li X Sun S. Hybrid task cascade for instance segmentation. In: Proceedings of the IEEE/CVF Conference on Computer Vision and Pattern Recognition. (2019). p. 4974–83.

[52] Wang X Kong T Shen C Jiang Y Li L. Solo: segmenting objects by locations. In: European Conference on Computer Vision. Cham: Springer (2020). p. 649–65.

[53] Bai M Urtasun R. Deep watershed transform for instance segmentation. In: Proceedings of the IEEE Conference on Computer Vision and Pattern Recognition. (2017). p. 5221–29. 34288972

[54] Xu K Guan K Peng J Luo Y Wang S. DeepMask: An Algorithm For Cloud and Cloud Shadow Detection in Optical Satellite Remote Sensing Images Using Deep Residual Network. arXiv [Preprint].arXiv:1911.03607 (2019).

[55] Carion N Massa F Synnaeve G Usunier N Kirillov A Zagoruyko S. End-to-end object detection with transformers. In: European Conference on Computer Vision. Cham: Springer (2020). p. 213–29.

[56] Prangemeier T Reich C Koeppl H. Attention-based transformers for instance segmentation of cells in microstructures. In: 2020 IEEE International Conference on Bioinformatics and Biomedicine (BIBM). IEEE (2020). p. 700–7.

[57] Hu J Cao L Lu Y Zhang S Wang Y Li K. ISTR: End-to-End Instance Segmentation With Transformers. arXiv [Preprint]. arXiv:2105.00637 (2021).

[58] Ren S He K Girshick R Sun J. Faster R-CNN: Towards real-time object detection with region proposal networks. Adv Neural Inf Process Syst. (2015) 28:91–9. 10.1109/TPAMI.2016.2577031 27295650

[59] Jain AK Chen H. Matching of dental X-ray images for human identification. Pattern Recognit. (2004) 37:1519–32. 10.1016/j.patcog.2003.12.016

[60] Fahmy GF Nassar DEM Said EH Chen H Nomir O Zhou J. Toward an automated dental identification system. J Electron Imaging. (2005) 14:043018. 10.1117/1.213531028584483

[61] Zhou J Abdel-Mottaleb M. A content-based system for human identification based on bitewing dental X-ray images. Pattern Recognit. (2005) 38:2132–42. 10.1016/j.patcog.2005.01.011

[62] Nomir O Abdel-Mottaleb M. A system for human identification from X-ray dental radiographs. Pattern Recognit. (2005) 38:1295–305. 10.1016/j.patcog.2004.12.01016119269

[63] Mahoor MH Abdel-Mottaleb M. Classification and numbering of teeth in dental bitewing images. Pattern Recognit. (2005) 38:577–86. 10.1016/j.patcog.2004.08.012

[64] Lin PL Lai YH Huang PW. An effective classification and numbering system for dental bitewing radiographs using teeth region and contour information. Pattern Recognit. (2010) 43:1380–92. 10.1016/j.patcog.2009.10.005

[65] Silva B Pinheiro L Oliveira L Pithon M. A study on tooth segmentation and numbering using end-to-end deep neural networks. In: 2020 33rd SIBGRAPI Conference on Graphics, Patterns and Images (SIBGRAPI). IEEE (2020). p. 164–71. 10.1109/SIBGRAPI51738.2020.00030

[66] Lurie A Tosoni GM Tsimikas J Walker Jr F. Recursive hierarchic segmentation analysis of bone mineral density changes on digital panoramic images. Oral Surg Oral Med Oral Pathol Oral Radiol. (2012) 113:549–58. e1. 10.1016/j.oooo.2011.10.002 22668434

[67] Tikhe SV Naik AM Bhide SD Saravanan T Kaliyamurthie KP. Algorithm to identify enamel caries and interproximal caries using dental digital radiographs. In: 2016 IEEE 6th International Conference on Advanced Computing (IACC). IEEE (2016). p. 225–8. 10.1109/IACC.2016.50

[68] Tuan TM. A cooperative semi-supervised fuzzy clustering framework for dental X-ray image segmentation. Expert Syst Appl. (2016) 46:380–93. 10.1016/j.eswa.2015.11.001

[69] Trivedi DN Kothari AM Shah S Nikunj S. Dental image matching by Canny algorithm for human identification. Int J Adv Comput Res. (2014) 4:985.

[70] Wirtz A Mirashi SG Wesarg S. Automatic teeth segmentation in panoramic X-ray images using a coupled shape model in combination with a neural network. In: International conference on medical image computing and computer-assisted intervention. Cham: Springer (2018). p. 712–9.

[71] Koch TL Perslev M Igel C Brandt SS. Accurate segmentation of dental panoramic radiographs with U-Nets. In: 2019 IEEE 16th International Symposium on Biomedical Imaging (ISBI 2019). Venice: IEEE (2019). p. 15–9. 10.1109/ISBI.2019.8759563

[72] Sivagami S Chitra P Kailash GSR Muralidharan SR. Unet architecture based dental panoramic image segmentation. In: 2020 International Conference on Wireless Communications Signal Processing and Networking (WiSPNET). IEEE (2020). p. 187–91.

[73] Choi J Eun H Kim C. Boosting proximal dental caries detection *via* combination of variational methods and convolutional neural network. J Signal Process Syst. (2018) 90:87–97. 10.1007/s11265-016-1214-6

[74] Cui W Zeng L Chong B Zhang Q. Toothpix: pixel-level tooth segmentation in panoramic X-Ray images based on generative adversarial networks. In: 2021 IEEE 18th International Symposium on Biomedical Imaging (ISBI). IEEE (2021). p. 1346–50.

[75] Zakirov A Ezhov M Gusarev M Alexandrovsky V Shumilov E. Dental pathology detection in 3D cone-beam CT. arXiv [Preprint].arXiv:1810.10309 (2018).

[76] Chen Y Du H Yun Z Yang S Dai Z Zhong L. Automatic segmentation of individual tooth in dental CBCT images from tooth surface map by a multi-task FCN. IEEE Access. (2020) 8:97296–309. 10.1109/ACCESS.2020.299179927295638

[77] Lee S Woo S Yu J Seo J Lee J Lee C. Automated CNN-Based tooth segmentation in cone-beam CT for dental implant planning. IEEE Access. (2020) 8:50507–18. 10.1109/ACCESS.2020.297582627295638

[78] Rao Y Wang Y Meng F Pu J Sun J Wang Q. Symmetric fully convolutional residual network with DCRF for accurate tooth segmentation. IEEE Access. (2020) 8:92028–38. 10.1109/ACCESS.2020.299459227295638

[79] Ezhov M Zakirov A Gusarev M. Coarse-to-fine volumetric segmentation of teeth in cone-beam CT. In: 2019 IEEE 16th International Symposium on Biomedical Imaging (ISBI 2019). Venice: IEEE (2019). p. 52–6.

[80] Zanjani FG Moin DA Verheij B Claessen F Cherici T Tan T. Deep learning approach to semantic segmentation in 3d point cloud intra-oral scans of teeth. In: International Conference on Medical Imaging with Deep Learning. PMLR (2019). p. 557–71.

[81] Jader G Fontineli J Ruiz M Abdalla K Pithon M Oliveira L. Deep instance segmentation of teeth in panoramic X-ray images. In: 2018 31st SIBGRAPI Conference on Graphics, Patterns and Images (SIBGRAPI). IEEE (2018). p. 400–7.

[82] Gurses A Oktay A B. Human Identification with Panoramic Dental Images using Mask R-CNN and SURF. In: 2020 5th International Conference on Computer Science and Engineering (UBMK). IEEE (2020). p. 232–7.

[83] Wu X Chen H Huang Y Guo H Qiu T Wang L. Center-sensitive and boundary-aware tooth instance segmentation and classification from cone-beam CT. In: 2020 IEEE 17th International Symposium on Biomedical Imaging (ISBI). IEEE (2020). p. 939–42.

[84] Cui Z Li C Wang W. ToothNet: automatic tooth instance segmentation and identification from cone beam CT images. In: Proceedings of the IEEE/CVF Conference on Computer Vision and Pattern Recognition. (2019). p. 6368–77.

[85] Zanjani FG Pourtaherian A Zinger S Moin DA Claessen F Cherici T. Mask-MCNet: tooth instance segmentation in 3D point clouds of intra-oral scans. Neurocomputing. (2021) 453:286–98. 10.1016/j.neucom.2020.06.145

[86] Silva G Oliveira L Pithon M. Automatic segmenting teeth in X-ray images: trends, a novel data set, benchmarking and future perspectives. Expert Syst Appl. (2018) 107:15–31. 10.1016/j.eswa.2018.04.001

[87] Kong Z Xiong F Zhang C Fu Z Zhang M Weng J. Automated maxillofacial segmentation in panoramic dental x-ray images using an efficient encoder-decoder network. IEEE Access. (2020) 8:207822–33. 10.1109/ACCESS.2020.303767727295638

[88] Li H Zhou J Zhou Y Chen J Gao F Xu Y. Automatic and interpretable model for periodontitis diagnosis in panoramic radiographs. In: International Conference on Medical Image Computing and Computer-Assisted Intervention. Cham: Springer (2020). p. 454–63.

[89] Egger J Pfarrkirchner B Gsaxner C Lindner L Schmalstieg D Wallner J. Fully convolutional mandible segmentation on a valid ground-truth dataset. In: 2018 40th Annual International Conference of the IEEE Engineering in Medicine and Biology Society (EMBC). Honolulu, HI: IEEE (2018). p. 656–60. 10.1109/EMBC.2018.8512458 30440482

[90] Zhang J Liu M Wang L Chen S Yuan P Li J. Joint craniomaxillofacial bone segmentation and landmark digitization by context-guided fully convolutional networks. In: International conference on medical image computing and computer-assisted intervention. Cham: Springer (2017). p. 720–8. 10.1007/978-3-319-66185-8_81 PMC5786437 29376150

[91] Torosdagli N Liberton D K Verma P Sincan M Lee J S Bagci U. Deep geodesic learning for segmentation and anatomical landmarking. IEEE Trans Med Imaging. (2018) 38:919–31. 10.1109/TMI.2018.2875814 30334750 PMC6475529

[92] Lian C Wang F Deng HH Wang L Xiao D Kuang T. Multi-task dynamic transformer network for concurrent bone segmentation and large-scale landmark localization with dental CBCT. In: International Conference on Medical Image Computing and Computer-Assisted Intervention. Cham: Springer (2020). p. 807–16. 10.1007/978-3-030-59719-1_78 PMC8687703 34935006

